# Cold Atmospheric-Pressure Plasma Caused Protein Damage in Methicillin-Resistant *Staphylococcus aureus* Cells in Biofilms

**DOI:** 10.3390/microorganisms9051072

**Published:** 2021-05-17

**Authors:** Li Guo, Lu Yang, Yu Qi, Gulimire Niyazi, Lingling Huang, Lu Gou, Zifeng Wang, Lei Zhang, Dingxin Liu, Xiaohua Wang, Hailan Chen, Michael G. Kong

**Affiliations:** 1 State Key Laboratory of Electrical Insulation and Power Equipment, Center for Plasma Biomedicine, Xi’an Jiaotong University, Xi’an 710049, China; qq460820339@stu.xjtu.edu.cn (Y.Q.); huangll@stu.xjtu.edu.cn (L.H.); zsdgsd@hotmail.com (Z.W.); xhw@mail.xjtu.edu.cn (X.W.); 2 School of Life Science and Technology, Xi’an Jiaotong University, Xi’an 710049, China; yanglu35@stu.xjtu.edu.cn (L.Y.); gulmira@stu.xjtu.edu.cn (G.N.); 3 School of Physics, Xi’an Jiaotong University, Xi’an 710049, China; goulul@stu.xjtu.edu.cn (L.G.); zhangleio@mail.xjtu.edu.cn (L.Z.); 4 Frank Reidy Center for Bioelectrics, Old Dominion University, Norfolk, VA 23508, USA; h1chen@odu.edu (H.C.); mglin5g@gmail.com (M.G.K.); 5 Department of Electrical and Computer Engineering, Old Dominion University, Norfolk, VA 23529, USA

**Keywords:** cold atmospheric-pressure plasma, biofilm, oxidative damage, reactive species, methicillin-resistant *Staphylococcus aureus*

## Abstract

Biofilms formed by multidrug-resistant bacteria are a major cause of hospital-acquired infections. Cold atmospheric-pressure plasma (CAP) is attractive for sterilization, especially to disrupt biofilms formed by multidrug-resistant bacteria. However, the underlying molecular mechanism is not clear. In this study, CAP effectively reduced the living cells in the biofilms formed by methicillin-resistant *Staphylococcus aureus*, and 6 min treatment with CAP reduced the *S. aureus* cells in biofilms by 3.5 log_10_. The treatment with CAP caused the polymerization of SaFtsZ and SaClpP proteins in the *S. aureus* cells of the biofilms. In vitro analysis demonstrated that recombinant SaFtsZ lost its self-assembly capability, and recombinant SaClpP lost its peptidase activity after 2 min of treatment with CAP. Mass spectrometry showed oxidative modifications of a cluster of peaks differing by 16 Da, 31 Da, 32 Da, 47 Da, 48 Da, 62 Da, and 78 Da, induced by reactive species of CAP. It is speculated that the oxidative damage to proteins in *S. aureus* cells was induced by CAP, which contributed to the reduction of biofilms. This study elucidates the biological effect of CAP on the proteins in bacterial cells of biofilms and provides a basis for the application of CAP in the disinfection of biofilms.

## 1. Introduction

*Staphylococcus aureus,* especially methicillin-resistant *S. aureus* (MRSA), is one of the most frequent causes of infections and generally forms biofilms on infected tissues [1,2,3,4,5]. Biofilms formed by aggregated microbial cells are surrounded by a self-produced extracellular polymeric matrix made of proteins, DNA, and polysaccharides and adhere to surfaces, such as those of living tissues [6,7]. The structure of the biofilm makes the biofilm-associated bacteria more resistant to the host immune defense system, antibiotics, and other antimicrobial agents [8,9]. Methicillin-resistant *S. aureus*, especially the multidrug-resistant strains, frequently exists in the hospital setting in the form of biofilms [10]. When the multidrug-resistant bacteria infect patients, they also exist in the wounds or other lesions in the form of biofilms [5,6]. The reduction of biofilms is critical for the prevention and treatment of infectious diseases caused by multidrug-resistant bacteria [11]. Therefore, the inactivation of biofilms formed by multidrug-resistant bacteria is a challenging issue in the therapy of infectious diseases.

Cold atmospheric-pressure plasma (CAP), known as the fourth state of matter, contains various reactive species, such as H_2_O_2_, ^1^O_2_, ^•^OH, and ^•^NO, as well as electrons, ions, and photons. The reactive species of CAP can effectively inactivate microorganisms and cells, which makes it attractive for a range of biomedical applications, such as sterilization and cancer treatment [12,13,14,15,16,17]. Planktonic methicillin-resistant *S. aureus* was fragile to the surface discharge plasma of CAP, but methicillin-resistant *S. aureus* usually exists in the form of biofilms in the environment [18]. Therefore, the inactivation effect of CAP on biofilms has been studied further. Xu et al. inactivated approximately 4 log_10_ *S. aureus* biofilms after treatment for 10 min with a He/O_2_ plasma jet, and Wang et al. inactivated approximately 0.5 log_10_ *S. aureus* cells in biofilms after 30 min of exposure to a N_2_ plasma jet [19,20]. Czapka et al. inactivated approximately 2.77 log_10_ *S. aureus* biofilms after treatment with dielectric barrier discharge (DBD) for 300 s [21]. However, the molecular mechanism in the bacterial cells in biofilms inactivated by CAP is poorly understood.

The filamentous temperature-sensitive Z (FtsZ) and ATP-dependent caseinolytic protease P subunit (ClpP) are both promising targets for antimicrobial agent development [22,23,24,25,26]. FtsZ is the major cytoskeleton protein of the cell division process in prokaryotes and assembles into short, single-stranded protofilaments with an average length of 120–250 nm in vitro, and into a ring structure in the middle of bacterial cells [22,27,28]. Mutation of Phe138 (F138A) causes a decrease in polymerization activity and guanosine triphosphatase (GTPase) activity [29]. The FtsZ protein is involved in cell division, and the inhibition of FtsZ assembly inhibits cell division [30,31]. FtsZ is essential in *S. aureus* and cannot be deleted [32]. FtsZ is also involved in biofilm formation, as demonstrated by a previous study showing the upregulation of the FtsZ protein in *Aggregatibacter actinomycetemcomitans* biofilms versus the planktonic state [33]. The protease ClpP is highly conserved and widely distributed in bacteria and is considered a regulator of the FtsZ protein [30,34]. The ClpP protease is involved in stress responses, the virulence of pathogenic bacteria, and antibiotic resistance [35,36,37,38]. The deletion or inhibition of ClpP proteases resulted in a decrease in persister *S. aureus* cells and affected virulence and stress response [6,39,40]. The crystal structures of ClpP proteases revealed that the SaClpP tetradecamer, which consisted of two heptameric rings, enclosed a large chamber containing 14 proteolytic active sites (Ser98-His123-Asp172) [41,42,43]. According to the findings of another previous study by our group, CAP can induce oxidative modification of peptides consisting of histidine and phenylalanine; therefore, CAP was proposed to have effects on FtsZ protein and ClpP proteases [44].

In this study, the molecular mechanism of CAP on biofilms was studied. The reactive species of CAP induced aggregation and inactivation of the FtsZ and ClpP proteins in the cells of *Staphylococcus aureus* biofilm. These results suggest that the reactive species induced protein damage, which contributed to the antibiofilm effects.

## 2. Materials and Methods

### 2.1. Biofilm Assay

The culture of *S. aureus* biofilms was performed as previously described [45]. *S. aureus* ATCC33591, a methicillin-resistant strain, was purchased from the American Type Culture Collection (ATCC). A single *S. aureus* ATCC33591 colony was grown in 4 mL of tryptic soy broth (TSB, Oxoid, Hampshire, UK) at 250 rpm at 37 °C overnight. The overnight *S. aureus* cultures were diluted 1:100 in TSB with 1% glucose. The single-side adhesive silica films (10 × 10 × 0.5 mm, purchased from Taobao) were attached on the bottom of wells of 24-well plates, covered with diluted *S. aureus*, and cultured at 37 °C for 3 days without medium renewal. The medium was carefully removed, and the silica films with biofilms were gently washed three times with saline solution (0.9% NaCl). Four replicates of biofilms were used for each treatment condition, and untreated biofilms and the biofilms treated with helium and 1% synthetic air without applying a voltage were used as control. After treatment, the biofilms were dispersed in 1 mL of saline in 1.5-mL Eppendorf tubes by sonication for 1 min and vortexing for 5 min. Serial dilutions of each biofilm were performed, and 10 μL of each dilution was spotted onto TSB plates and incubated overnight at 37 °C.

### 2.2. CAP Device and CAP Treatments

**Figure 1:**
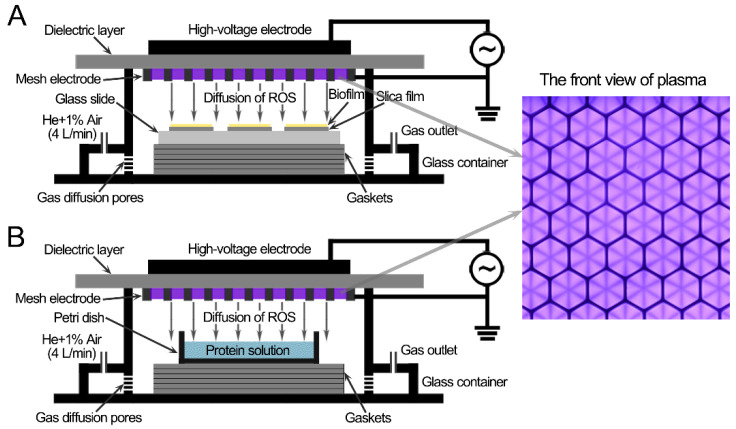
Schematic diagram of the *S. aureus* biofilms (**A**) and the protein solutions (**B**) treated with the surface discharge of cold atmospheric-pressure plasma.

The CAP device was similar to the previous study [45]. The surface discharge structure of the CAP consisted of a high-voltage plane electrode, a liquid-facing grounded mesh electrode with a hexagonal shape, and a dielectric layer (made of polytetrafluoroethylene) sandwiched between the two electrodes (Figure 1). The surface discharge plasma (40 mm × 40 mm) was generated when a sinusoidal high voltage with a frequency of 20 kHz was applied. The applied voltage and discharge current were measured with a high-voltage probe (Tektronix P6015A, Portland, OR, USA) and a current probe (Pearson 2877, Palo Alto, CA, USA), respectively. The discharge power was obtained by integrating the applied voltage and discharge current demonstrated by an oscilloscope (Tektronix, MDO3054, Portland, OR, USA). The discharge power was controlled at approximately 3.2 W (the discharge power density of 0.2 W/cm^2^) with good mesh-to-mesh homogeneity through regulating the applied voltage. The values of the peak-to-peak voltage and discharge current were approximately 7.44 kV and 40.8 mA, respectively.

*S. aureus* biofilms on sterilized glass slides (a diameter of 30 mm) or protein solutions (150 μL) in a Petri dish (a diameter of 15 mm), which was smaller in size than the CAP, were placed under the CAP, and the air gap between the CAP and the surface of the biofilms or liquid surfaces was 8 ± 0.2 mm (Figure 1). The CAP treatment system was housed in a sealed organic glass box, and the gas flow of helium and 1% synthetic air (79% N_2_ + 21% O_2_) flowed into the organic glass box at a constant rate of 4 L/min, controlled by the mass flow controller (MFC, Sevenstar, Beijing, China), diffused through pores at the bottom part, and then spread upward to the underneath of the surface plasma. The flow rate at the outlet was less than 1 L/min measured by a rotameter, which indicated that most of the gas leaked through the small gaps that were not completely sealed to prevent the increase in pressure in the chamber (Figure 1). Synthetic air was used as the source of reactive species, while helium was used to enhance the production efficiency of those species as well as their flux on the treated samples via diffusion. The ambient temperature of the gas chamber and the temperature of samples were measured using a thermocouple, and both the temperatures were lower than 35 °C after 6 min of CAP treatment at a room temperature of 28 °C.

### 2.3. Protein Extraction and Detection

The *S. aureus* cells from CAP-treated and untreated biofilms were resuspended in solubilization buffer (50 mM imidazole, 50 mM NaCl, 2 mM 6-aminohexanoic acid, 1 mM EDTA, and 1% dodecylmaltoside; pH 7.0), and lysis was performed by bead beating in a MiniBeadbeater-16 (Biospec, Bartlesville, OK, USA). After centrifugation, the supernatants were transferred, and protein concentrations were determined by the BCA Protein Assay Kit (Beyotime, Shanghai, China) using bovine serum albumin (BSA) as the standard. The soluble proteins (10 μg) were mixed with Laemmli buffer (Bio-Rad, Hercules, CA, USA) and subjected to 15% SDS-PAGE. The gel was stained with Coomassie Brilliant Blue G-250 or transferred to PVDF membranes and subjected to immunoblotting.

### 2.4. SaFtsZ and SaClpP Protein Expression and Purification

The genes encoding FtsZ (ORF: SACOL1199) and ClpP (ORF: SACOL0833) were amplified from the genomic DNA of *S. aureus* ATCC33591 by PCR using Pfu DNA polymerase (Stratagene, La Jolla, CA, USA) (for primer sequences, see Table 1). The PCR product was inserted into the NdeI/XhoI sites of the expression vector pET-28a (for His-tagged SaFtsZ) or pET-32a (for His-tagged and untagged SaClpP) (Novagen, Darmstadt, Germany). Expression plasmids were transformed into *E. coli* BL21(DE3) cells. The cells were grown and induced by the addition of isopropyl-β-D-thiogalactopyranoside (IPTG). For His-tagged SaFtsZ and SaClpP, the induced cells were suspended in lysis buffer containing 50 mM Tris-Cl (pH 7.5) and 500 mM NaCl and then lysed at 4 °C by sonication. After centrifugation at 16,000× *g* for 20 min to remove the insoluble material, the supernatant was loaded onto a Histrap column (1 mL; GE Healthcare, Marlborough, MA, USA), and the protein was eluted with a single gradient of 0.5 M imidazole in lysis buffer. Fractions containing SaFtsZ or SaClpP were pooled and dialyzed against 50 mM HEPES (pH 7.5), 5 mM MgCl_2_, and 100 mM KCl. For untagged SaClpP, the induced cells were suspended in lysis buffer containing 50 mM Tris-Cl (pH 7.5), 100 mM NaCl, and 1 mM dithiothreitol (DTT) and lysed at 4 °C by sonication. After centrifugation at 16,000× *g* for 20 min to remove the insoluble material, the supernatant was loaded onto a Hitrap Capto Q column (5 mL; GE Healthcare), and the protein was eluted with a single gradient of 1 M NaCl in lysis buffer. The Q column fractions containing SaClpP were pooled and loaded onto a Superdex G200 column (24 mL; GE Healthcare, Marlborough, MA, USA) equilibrated with 50 mM Tris-Cl (pH 7.5), 100 mM NaCl, and 1 mM DTT. Fractions containing SaClpP were pooled and dialyzed against 10 mM Tris-Cl (pH 7.5), 1 mM EDTA, and 1 mM DTT. Protein concentrations were determined by a BCA Protein Assay Kit (Beyotime, Shanghai, China) using bovine serum albumin (BSA) as the standard.

### 2.5. Immunoblotting

Anti-SaFtsZ and anti-SaClpP antibodies were raised in rabbits using purified His-tagged SaFtsZ and SaClpP. The blots were blocked in 5% milk/Tris-buffered saline with 0.5% Tween (TBST) at 4 °C overnight with gentle agitation. After incubation with anti-SaFtsZ or anti-SaClpP antibodies for 2 h at room temperature with gentle agitation, the blots were washed four times (10 min per wash) with TBST at room temperature. The blots were incubated with Dylight649 Goat Anti-Rabbit IgG (Abbkine, Wuhan, China) for 1 h at room temperature with gentle agitation and washed again as described above. The blots were detected using a Typhoon FLA9500 biomolecular imager (GE Healthcare, Marlborough, MA, USA).

### 2.6. Blue Native Polyacrylamide Gel Electrophoresis

The recombinant SaFtsZ and untagged SaClpP proteins (1 mg/mL) in 10 mM Tris-Cl (pH 7.5) and 1 mM EDTA were treated with CAP for 2 min, as shown in Figure 1B. The soluble proteins (20 μg) from the CAP-treated and untreated biofilms were mixed with 4× loading buffer (200 mM imidazole, 200 mM NaCl, 20% glycerol, and 0.02% Ponceau S) and electrophoresed through a 10–20% (for SaFtsZ) or 4–15% (for SaClpP) gradient blue native polyacrylamide gel [46]. The gel was stained with Coomassie Brilliant Blue R-250 or subjected to immunoblotting. The gel for immunoblotting was washed with electroblotting buffer (25 mM Tris-Cl, pH 7.5, 250 mM glycine, and 0.1% sodium dodecyl sulfate (SDS)) for 30 min and transferred to a PVDF membrane using the electroblotting buffer for 3 h at 4 °C with a current of 150 mA. After transfer, methanol was used to destain the background of the PVDF membrane. Then, the PVDF membranes were subjected to immunoblotting.

### 2.7. In Vitro Assembly Assay of SaFtsZ

The SaFtsZ proteins treated with the CAP, treated with the mixture of H_2_O_2_ + NO_2_^−^ + NO_3_^−^ (saline with 250 μM H_2_O_2_ + 125 μM NO_2_^−^ + 375 μM NO_3_^−^), or left untreated were incubated with 2 mM guanosine triphosphate (GTP) in HMK buffer (50 mM HEPES-OH, pH 7.5, 5 mM MgAc, and 100 mM KCl) for 10 min at 37 °C. Then, the SaFtsZ proteins were diluted five times with HMK buffer. For negative staining, an aliquot (~4 μL) of proteins was dropped onto a glow-discharged thin carbon-coated 200-mesh grid (Carbon Gilder Finder Grids, Cu, F1 UL) and kept for 1 min at 22 °C. Then, the excess solution was removed by touching a piece of filter paper to the back of the grid. The grid was washed by briefly touching the surface of the grid with a drop (~30 μL) of deionized water on Parafilm and then blotted dry with filter paper. The touching and blotting steps were repeated once. Two successive drops (~25 μL/drop) of 1% (*w/v*) uranyl acetate (UA) solution were applied to Parafilm, and the excess solution was removed by blotting similarly. The grid remained in contact with the last UA drop with the sample side down for 1 min in the dark before the excess stain was removed, and the sample was air-dried at room temperature. The stained samples were examined using an FEI Talos F200C transmission electron microscope operating at 200 kV at 57,000× magnification.

### 2.8. Enzymatic Activity Assay of SaClpP

Peptidase activity was detected as described previously using the model fluorogenic substrate dipeptide *N*-succinyl-Leu-Tyr-7-amido-4-methylcoumarin (Suc-LY-AMC) (MP Biomedicals, Santa Ana, CA, USA) [47]. The CAP-treated, H_2_O_2_ + NO_2_^−^ + NO_3_^−^ treated, or untreated SaClpP (5 μM monomer) was incubated with Suc-LY-AMC (250 μM) in 50 mM HEPES-OH (pH 7.5) and 100 mM NaCl. The hydrolysis of the labeled peptide led to the release of fluorescent AMC. The fluorescence was measured on a microplate reader (Thermo Scientific Varioskan Flash, Waltham, MA, USA) at 37 °C for 30 cycles every 60 s using an excitation wavelength of 340 nm and an emission wavelength of 450 nm, as described previously [48].

### 2.9. Mass Spectrometry of SaClpP

The CAP-treated and untreated SaClpP (5 μg) were subjected to SDS-PAGE (15%) and visualized by staining with Coomassie Brilliant Blue R-250. The protein bands were excised, and the protein was digested in-gel with trypsin, as described previously [49]. The digested products were dissolved in 0.1% formic acid and purified with ZipTipC18 pipette tips (Millipore, Darmstadt, Germany) according to the manufacturer’s procedure. The tryptic peptides were identified using an Orbitrap Fusion Tribrid mass spectrometer (ThermoFisher, Waltham, MA, USA) coupled to an EASY-nLC 1000 UPLC system (ThermoFisher, Waltham, MA, USA).

## 3. Results

### 3.1. Inactivation of CAP Treatment on Biofilms

**Figure 2:**
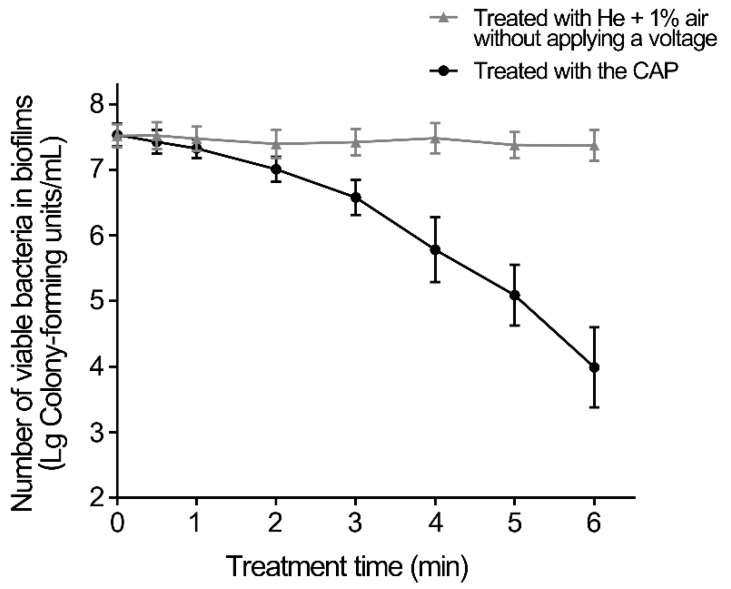
Inactivation effect on *S. aureus* biofilms of CAP. The biofilms were treated with CAP for the indicated times, and the CAP-treated and untreated biofilms were dispersed in 1 mL saline by sonication and vortexing. Serial dilutions of each biofilm were performed, and 10 μL of each dilution was spotted onto TSB plates and incubated overnight at 37 °C. Error bars represent the standard deviation (SD).

The inactivation effect of CAP on *S. aureus* biofilms was first determined (Figure 2). The CAP treatment for 2 min decreased the number of living *S. aureus* cells by approximately 0.5 log_10_ in biofilms, while that for 4 min decreased them by less than 2 log_10_. After the biofilms were treated with CAP for 6 min, the living cells in the biofilms were reduced by approximately 3.5 log_10_ in the biofilms. Therefore, CAP treatment can effectively reduce the living cells in *S. aureus* biofilms.

### 3.2. CAP Treatment Causes Polymerization of the SaFtsZ and SaClpP and Induces Oxidative Modification of Proteins

**Figure 3:**
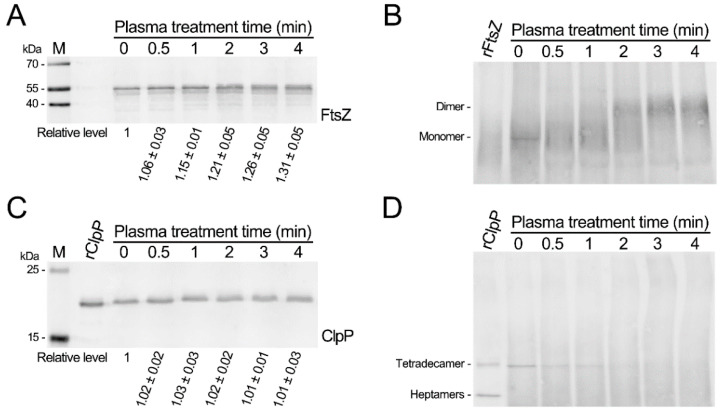
Polymerization of SaFtsZ and SaClpP in *S. aureus* biofilms induced by plasma treatment. (**A**) Immunoblotting of SaFtsZ under denaturing conditions. (**B**) Immunoblotting of SaFtsZ under nondenaturing conditions. (**C**) Immunoblotting of SaClpP under denaturing conditions. (**D**) Immunoblotting of SaClpP under nondenaturing conditions. The plasma-treated and untreated biofilms were dispersed and lysed, and then the soluble proteins were extracted and subjected to SDS-PAGE or blue native PAGE. The SaFtsZ and SaClpP proteins were detected by immunoblotting using anti-SaFtsZ and anti-SaClpP antibodies, respectively.

To investigate the molecular mechanisms underlying the inactivation of CAP treatment, the soluble proteins from the CAP-treated and untreated *S. aureus* biofilms separated by both SDS-PAGE and blue-native gels and the SaFtsZ and SaClpP proteins were detected using immunoblotting. The SaFtsZ protein of the untreated biofilms exhibited a single band, whereas that of the CAP-treated biofilms exhibited two close bands (Figure 3A and Figure S1). The emerging band migrated slightly more slowly than the main band and increased with the time of CAP treatment. Then, blue native gels were employed to identify changes in the SaFtsZ protein. Compared with the SaFtsZ protein of the untreated biofilms, the SaFtsZ protein of the biofilms treated with the CAP for 1 min started to form larger polymers, and the SaFtsZ protein of the biofilms treated with the CAP for 3 min formed larger polymers (Figure 3B). The bands of the SaClpP protein from the biofilms after the CAP treatment for 1 to 4 min migrated slightly more slowly than those from the untreated biofilms (Figure 3C). The bands of the SaClpP protein became dispersive and fuzzy in blue native gels from biofilms treated with the CAP for 1 min compared with untreated biofilms. The SaClpP protein was hardly detectable after the CAP treatment for 2 min to 4 min, and the smear of larger polymers became more intense (Figure 3D). These results indicate that the CAP treatment of *S. aureus* biofilms caused the polymerization of the SaFtsZ and SaClpP proteins.

### 3.3. CAP Treatment Causes Inactivation of the SaFtsZ and SaClpP Proteins

**Figure 4:**
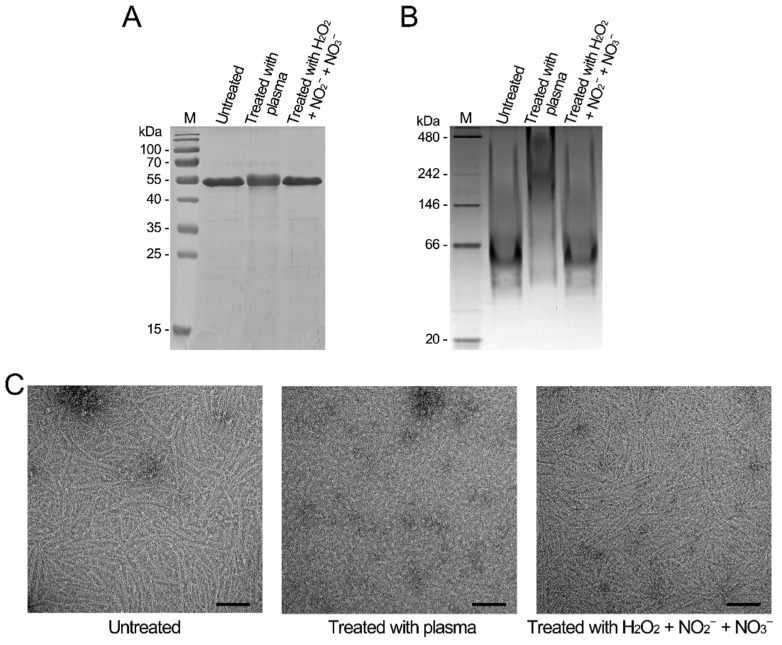
Inactivation of SaFtsZ by plasma treatment in vitro. (**A**) The plasma-treated, H_2_O_2_ + NO_2_^−^ + NO_3_^−^-treated, and untreated SaFtsZ proteins separated by SDS-PAGE. (**B**) The plasma-treated, H_2_O_2_ + NO_2_^−^ + NO_3_^−^-treated, and untreated SaFtsZ proteins separated by blue native PAGE. The SDS-PAGE or blue native PAGE was stained with Coomassie Brilliant Blue R-250. M, molecular weight standards with molecular weights indicated. (**C**) Self-assembly of SaFtsZ proteins. The plasma-treated, H_2_O_2_ + NO_2_^−^ + NO_3_^−^-treated, and untreated SaFtsZ proteins were self-assembled in the presence of GTP. Then, the samples were negatively stained and examined using transmission electron microscopy (TEM).

To analyze the biological effects of the CAP treatment on proteins, the recombinant SaFtsZ and SaClpP proteins were treated with the CAP. The SaFtsZ and SaClpP proteins in *S. aureus* cells were entirely damaged after treatment by CAP for 2 min; therefore, the treatment time of recombinant proteins was 2 min. Based on the previous study, three kinds of long-lived reactive species, namely, H_2_O_2_, NO_2_^−^, and NO_3_^−^, were generated in the same CAP, with concentrations of 53.3 μM, 25.4 μM, and 148.0 μM, respectively [38]. Then, a mixed solution with higher concentrations of H_2_O_2_ (100 μM), NO_2_^−^ (100 μM), and NO_3_^−^ (500 μM) was also employed for comparison. After the CAP treatment, the denatured SaFtsZ protein migrated slightly more slowly than the untreated SaFtsZ protein (Figure 4A). The untreated SaFtsZ protein was a monomer, and the CAP-treated SaFtsZ protein formed larger polymers (Figure 4B). To further evaluate the effect on the proteins, the self-assembling capacity of SaFtsZ protein was analyzed by electron microscopy (EM). The CAP-treated SaFtsZ protein lost its self-assembly capability, whereas the untreated SaFtsZ protein self-assembled into protofilaments in the presence of GTP (Figure 4C).

**Figure 5:**
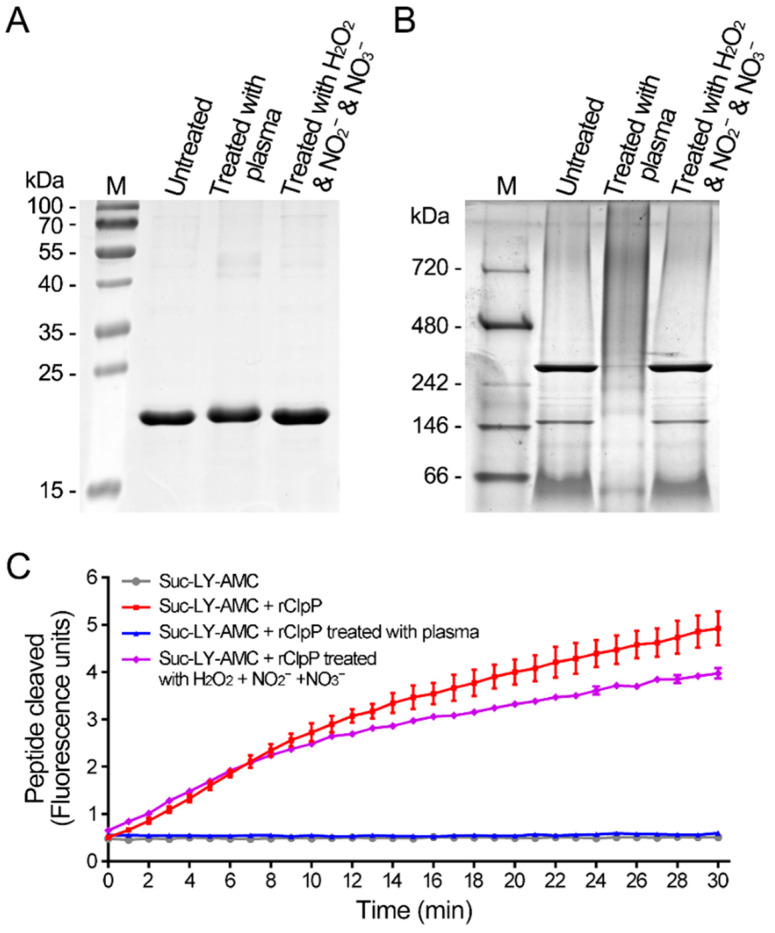
Inactivation of SaClpP by plasma treatment in vitro. (**A**) The plasma-treated, H_2_O_2_ + NO_2_^−^ + NO_3_^−^-treated, and untreated SaClpP proteins separated by SDS-PAGE. (**B**) The plasma-treated, H_2_O_2_ + NO_2_^−^ + NO_3_^−^-treated, and untreated SaClpP proteins separated by blue native PAGE. The SDS-PAGE or blue native PAGE was stained with Coomassie Brilliant Blue R-250. M, molecular weight standards with molecular weights indicated. (**C**) Peptidase activity of SaClpP proteins. The time courses of hydrolysis of the fluorogenic peptide *N*-succinyl-Leu-Tyr-7-amido-4-methylcoumarin by the plasma-treated, H_2_O_2_ + NO_2_^−^ + NO_3_^−^ treated, and untreated SaClpP proteins at 37 °C were measured. Error bars represent the standard deviation (SD).

The denatured CAP-treated SaClpP protein migrated more slowly than the untreated SaClpP protein (Figure 5A). The untreated SaClpP protein exhibited tetradecamers and heptamers, and the CAP-treated SaClpP protein became a larger smear probably formed by large polymers (Figure 5B). To further evaluate the effect on proteins, peptidase activity against peptide substrates was tested. The cleavage of the dipeptide (*N*-succinyl-Leu-Tyr-7-amido-4-methylcoumarin) by CAP-treated SaClpP was hardly detectable, suggesting that the peptidase activity of the SaClpP protein was inactivated by the CAP treatment (Figure 5C). These results indicated that the CAP treatment caused polymerization and inactivated the activity of the SaFtsZ and SaClpP proteins in vitro.

### 3.4. CAP Induces Oxidative Modification in Proteins

**Figure 6:**
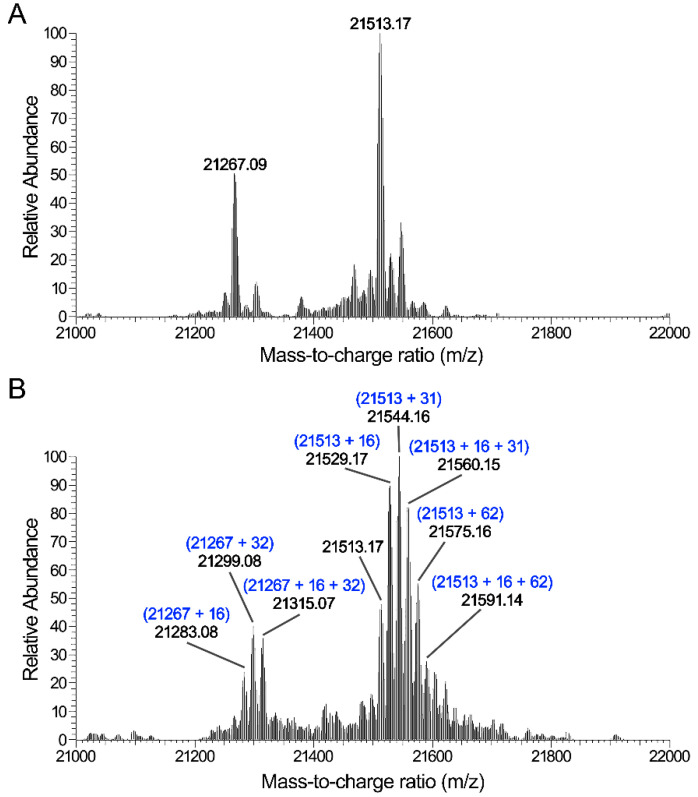
Identification of the modifications of SaClpP induced by plasma treatment. (**A**) Mass spectrum of untreated SaClpP. (**B**) Mass spectrum of the plasma-treated SaClpP. A cluster of peaks with mass differences of 16, 31, 32, or 62 Da are shown.

To elucidate the mechanism underlying the effect of the CAP treatment on proteins, the SaClpP proteins were analyzed by mass spectrometry. The mass weights of untreated SaClpP proteins were 21,513.17 (theoretical Mr: 21,513.57) and 21,267.09 with two amino acid deletions of the N-terminus (Figure 6A). The CAP-treated SaClpP appeared to be modified at multiple sites: a cluster of peaks differing by 16 Da, 31 Da, 32 Da, 47 Da, 48 Da, 62 Da, and 78 Da were detected (Figure 6B). The shifts of 16 Da and 32 Da were consistent with the hydroxy group (ROH) and the hydroperoxide group (ROOH) or two hydroxy groups, respectively. The shift of 31 Da corresponded to ROO- or a combination of one RO- and one hydroxy group, and the shift of 62 Da corresponded to the combination of two ROO- groups, one RO-OR (+30 Da) group and one hydroperoxide group, or two hydroxy groups. Mass fingerprinting showed that the SaClpP protein was oxidatively modified at multiple sites (Table S1 and Figure S2). These results demonstrate that the reactive oxygen species from the CAP induced oxidative modification of the SaClpP protein.

## 4. Discussion

CAP based on the electrical method can efficiently inactivate bacteria and reduce biofilms. The SaFtsZ and SaClpP proteins could be modified and inactivated by the CAP treatment in both in vitro and *S. aureus* cells, which contributed to the reduction of biofilms by CAP. The understanding of the molecular mechanism of this process is helpful in optimizing the control of CAP and guiding the application of CAP.

CAP contains various reactive species, such as long-lived H_2_O_2_, NO_2_^−^, and NO_3_^−^ and short-lived ^•^OH, ^1^O_2_, ^•^NO, and ONOO^−^, as previously described [18,44]. The mixture of three long-lived species, namely, H_2_O_2_, NO_2_^−^, and NO_3_^−^, exhibited little effect on SaFtsZ and SaClpP proteins, which demonstrated that the short-lived species were critical in the process. The reactive species penetrated bacterial cells in biofilms and induced the polymerization of the SaFtsZ and SaClpP proteins. A previous study also found that CAP-induced polymerization of GFP-tagged proteins caused protein denaturation and insolubility in *Saccharomyces cerevisiae* [50]. In this study, the polymerized proteins were in the soluble fraction, and this effect could be reversed by the reducing agent. These effects seemed to be similar to the effects of disulfide bond formation, and the reactive oxygen species could also cause cysteine to form disulfide bonds. However, the SaFtsZ protein did not contain cysteine, and further mass spectrometry analysis demonstrated that the oxidative modifications of the SaClpP protein were mainly induced by the CAP treatment. Therefore, oxidative modifications, such as ROOR, occurred between proteins and caused protein polymerization. ^1^O_2_ and O_2_^•−^ are both generated by CAP and can rapidly and selectively react with amino acids, efficiently yielding hydroperoxide groups [44,51,52,53,54]. Therefore, the reactive species of CAP reacted with proteins and caused the polymerization and function loss of proteins through oxidative modifications.

The treatment of CAP exhibited similar effects on the recombinant SaFtsZ and SaClpP proteins and the SaFtsZ and SaClpP proteins from *S. aureus* biofilms. However, the in vitro analysis could not fully reflect the effects in bacterial cells for two reasons. The first reason is that the reactive species need to cross the cell walls and membranes into the cells and then react with the proteins in the cells [55,56]. Not all of the reactive species of CAP could enter into the cells. The second reason is that the reactive species entering into the cells were affected by the enzymes in the cell or reacted with the reactive species produced by the cells [57]. The types and amounts of reactive species in the cells would change. In the in vitro treatment, the reactive species directly reacted with the recombinant proteins without the obstacle of the cell wall and membrane and the effects of the intracellular enzymes.

It is inevitable that the reactive species of CAP will react with other proteins in *S. aureus* cells and cause damage due to the non-selectivity and high reactivity of radical species [52]. Previous studies demonstrated that CAP induced double-stranded and single-stranded breaks in DNA and crosslinks between DNA and proteins in *Escherichia coli* cells [44,58]. Therefore, the reactive species of CAP caused oxidative damage on DNA and various proteins, including SaFtsZ and SaClpP, and the damages synergistically caused the death of *S. aureus* cells in the biofilm.

**Figure 7:**
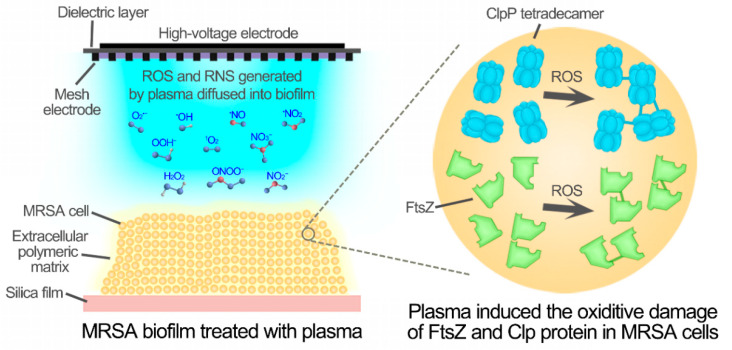
The aggregation and inactivation of SaFtsZ and SaClpP induced by the reactive species of CAP.

Based on these results, a model of CAP treatment for biofilm eradication was proposed (Figure 7). Reactive oxygen species generated by plasma, such as O_2_^•−^, diffused into biofilms and entered into *S. aureus* cells. The reactive oxygen species reacted with proteins, such as SaFtsZ and SaClpP, and induced oxidative modifications that caused polymerization and functional loss of proteins. Inactivation of SaFtsZ and SaClpP blocked the growth and division of *S. aureus* cells, resulting in the death and eradication of biofilms. Therefore, cold atmospheric-pressure plasma could be developed and used in strategies for both the disinfection of biofilms and therapy for wound infections.

## Figures and Tables

**Table 1:** The primers used in this study.

Designation	Sequence (5′-3′)
*ftsZ* up	ATCATGCCATGGTAGAATTTGAACAAGGATTTA
*ftsZ* His-tag down	ATACCGCTCGAGACGTCTTGTTCTTCTTGAACGTC
*clpP* up	ATGGGAATTCCATATGAATTTAATTCCTACAGTTATTGAAAC
*clpP* His-tag down	ATACCGCTCGAGTTTTGTTTCAGGTACCATCACTTC
*clpP* down	ATACCGCTCGAGTTATTTTGTTTCAGGTACCATCACTTC

## Data Availability

The mass spectrometry data have been deposited to the ProteomeXchange Consortium via the PRIDE [59] partner repository with the dataset identifier PXD023352 and 10.6019/PXD023352.

## Supplementary Materials

The following are available online at https://www.mdpi.com/article/10.3390/microorganisms9051072/s1.
