# Supplementary material for: Cold Atmospheric-Pressure Plasma Caused Protein Damage in Methicillin-Resistant Staphylococcus aureus Cells in Biofilms
Source: Microorganisms. 2021 May 17;9(5):1072. doi: 10.3390/microorganisms9051072 (PMC8156483; doi:10.3390/microorganisms9051072)
Supplement: Supplementary file 1 [file microorganisms-09-01072-s001.zip › microorganisms-1212520-supplementary.pdf]

Supplementary Information

Supplementary Table

Supplementary Table 1. Tryptic fragments of plasma-treated and untreated SaClpP protein identified by mass spectrometry. The modification fragments in plasma-treated SaClpP protein were indicated in blue.

Amino acid residue No.	Calculated mass (Da)	Measured mass (Da)	Sequences of identified peptides	Spectrum count
**Plasma-treated SaClpP**				
1–13	1500.79	1500.80	MNLIPTVIETTNR	62
1–13	1516.80	1516.79	M(O)NLIPTVIETTNR	31
1–16	1842.97	1842.96	MNLIPTVIETTNRGER	14
1–16	1858.96	1858.96	M(O)NLIPTVIETTNRGER	8
2–13	1369.76	1369.76	NLIPTVIETTNR	10
2–16	1711.93	1711.92	NLIPTVIETTNRGER	1
17–23	886.42	886.42	AYDIYSR	14
**17–23**	**902.42**	**902.42**	**AYDIY(O)SR**	**3**
112–145	3636.92	3636.92	FALPNAEVMIHQPLGGAQGQATEIEIAANHILK	3
**112–145**	**3651.92**	**3651.91**	**FALPNAEVMIHQPLGGAQ(–O–)GQATEIEIAANHILK**	**1**
**112–145**	**3652.92**	**3652.91**	**FALPNAEVMIHQPLGGAQ(O)GQATEIEIAANHILK**	**1**
113–145	3480.83	3480.82	FALPNAEVMIHQPLGGAQGQATEIEIAANHILK	9
113–145	3496.82	3496.81	FALPNAEVMIHQPLGGAQ(O)GQATEIEIAANHILK	5
**113–145**	**3512.81**	**3512.81**	**FALPNAEVMIHQPLGGA(OO)QGQATEIEIAANHILK**	**1**
113–147	3737.98	3737.97	FALPNAEVMIHQPLGGAQGQATEIEIAANHILKTR	1
**113–147**	**3753.97**	**3753.96**	**FALPNAEVMIH(O)QPLGGAQGQATEIEIAANHILKTR**	**1**
158–164	761.39	761.39	TGQSIEK	2
165–181	1992.98	1992.98	IQKDTDRDNFLTAEEAK	14
168–181	1623.74	1623.74	DTDRDNFLTAEEAK	69
**168–181**	**1639.74**	**1639.73**	**DTDRDNF(O)LTAEEAK**	**10**
**168– 181**	**1654.71**	**1654.72**	**DTDRDNFLTAEEAK(–OO–)**	**1**
168–195	3227.53	3227.52	DTDRDNFLTAEEAKEYGLIDEVMVPETK	19
168–195	3272.51	3272.48	DTDRDNFLTAEEAKEYGLIDEVMVPETK(3 –O–)	2
**168–195**	**3243.52**	**3243.51**	**DTDRDNFLTAEEAKEY(O)GLIDEVMVPETK**	**24**
**168– 195**	**3259.52**	**3259.51**	**DTDRDNFLTAEEAKEYGLIDEVM(OO)VPETK**	**2**
172–181	1136.54	1136.53	DNFLTAEEAK	7
172–195	1621.80	1621.79	DNFLTAEEAKEYGLIDEVMVPETK	1
172–195	2740.32	2740.32	DNFLTAEEAKEYGLIDEVM(O)VPETK	1
182–195	1621.80	1621.79	EYGLIDEVMVPETK	27
182–195	1637.79	1637.79	EYGLIDEVM(O)VPETK	49

182–195	1653.79	1653.78	EYGLIDEVMVPETK(OO)	1
**Untreated SaClpP**				
1–13	1500.79	1500.80	MNLIPTVIETTNR	74
1–13	1516.80	1516.79	M(O)NLIPTVIETTNR	16
1–16	1842.97	1842.96	MNLIPTVIETTNRGER	17
1–16	1858.96	1858.96	M(O)NLIPTVIETTNRGER	7
2–13	1369.76	1369.76	NLIPTVIETTNR	10
2–16	1711.92	1711.92	NLIPTVIETTNRGER	2
17–23	886.42	886.42	AYDIYSR	6
113–145	3480.83	3480.82	FALPNAEVMIHQPLGGAQGQATEIEIAANHILK	2
113–145	3496.83	3496.81	FALPNAEVM(O)IHQPLGGAQGQATEIEIAANHILK	1
158–164	761.39	761.39	TGQSIEK	1
158–167	11.30.63	1130.63	TGQSIEKIQK	1
165–181	1992.98	1992.98	IQKDTDRDNFLTAEEAK	8
168–181	1623.74	1623.74	DTDRDNFLTAEEAK	48
168–195	3227.53	3227.52	DTDRDNFLTAEEAKEYGLIDEVMVPETK	3
172–181	1136.54	1136.53	DNFLTAEEAK	6
182–195	1621.80	1621.79	EYGLIDEVMVPETK	62
182–195	1637.79	1637.79	EYGLIDEVM(O)VPETK	37

Supplementary Figures

Supplementary Figure 1. SDS-PAGE of soluble proteins from *S. aureus* biofilms treated with LTGP or untreated.

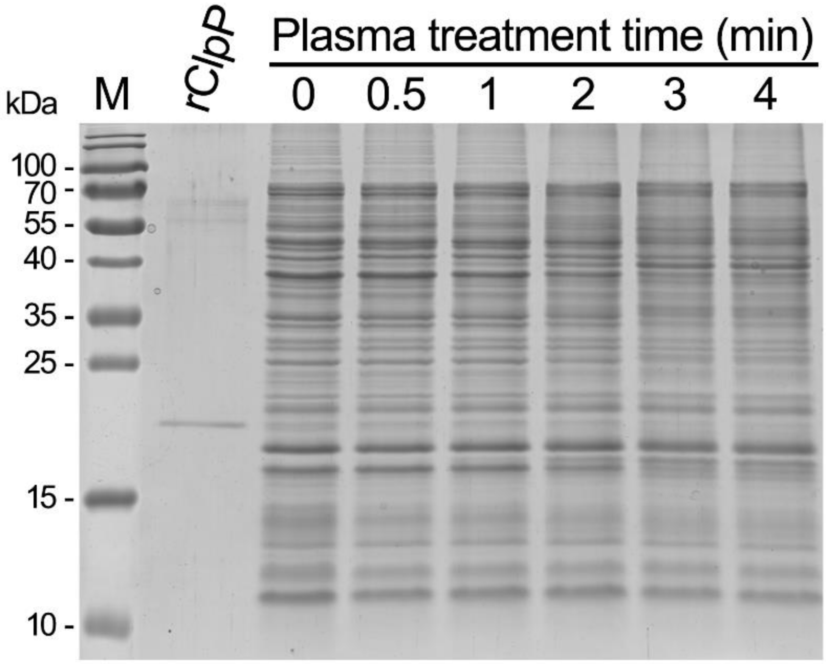

Supplementary Figure 2. The MS/MS spectrum of three oxidatively modified peptides derived from the tryptic digestion of LTGP-treated SaClpP protein.

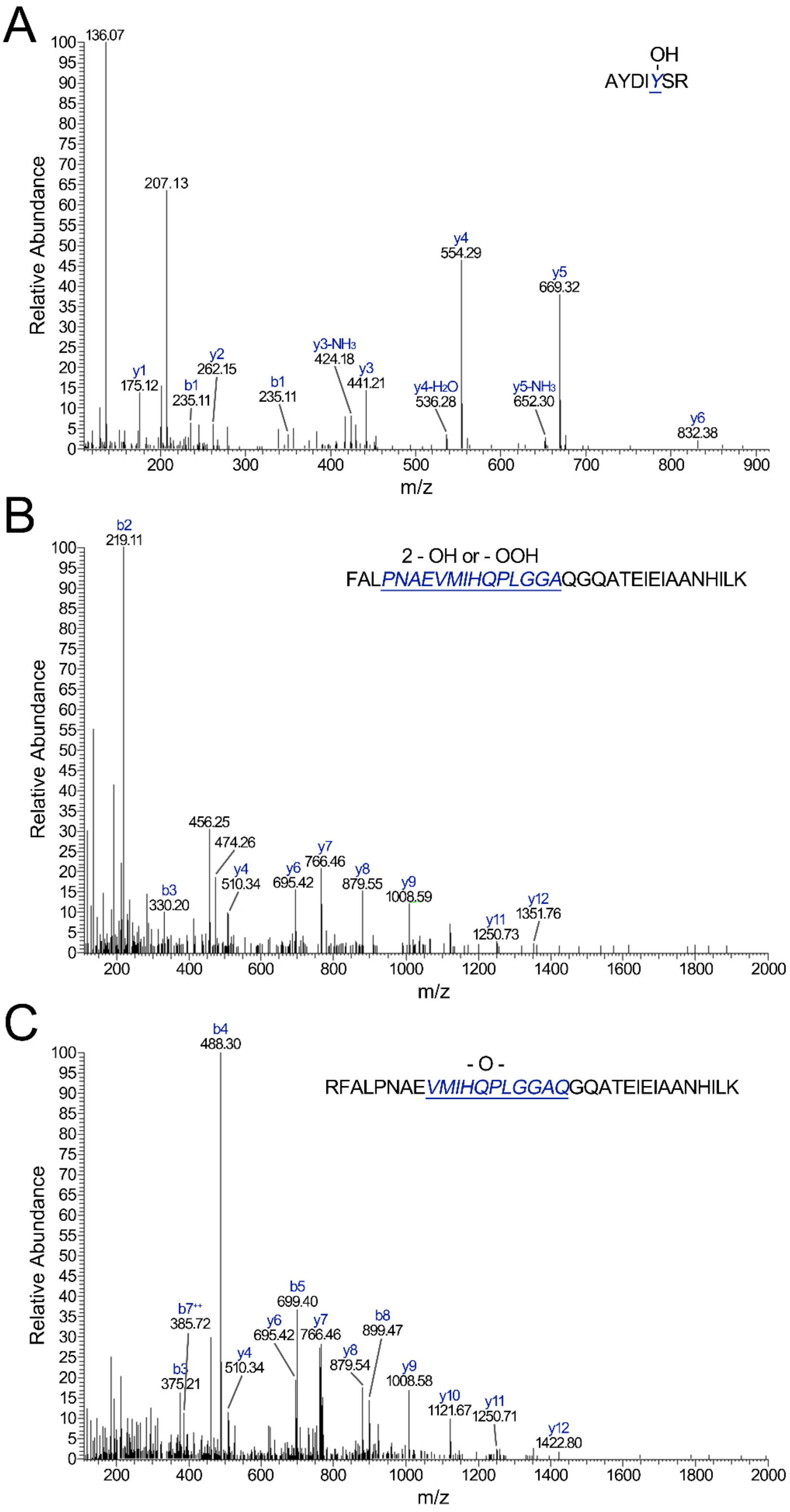

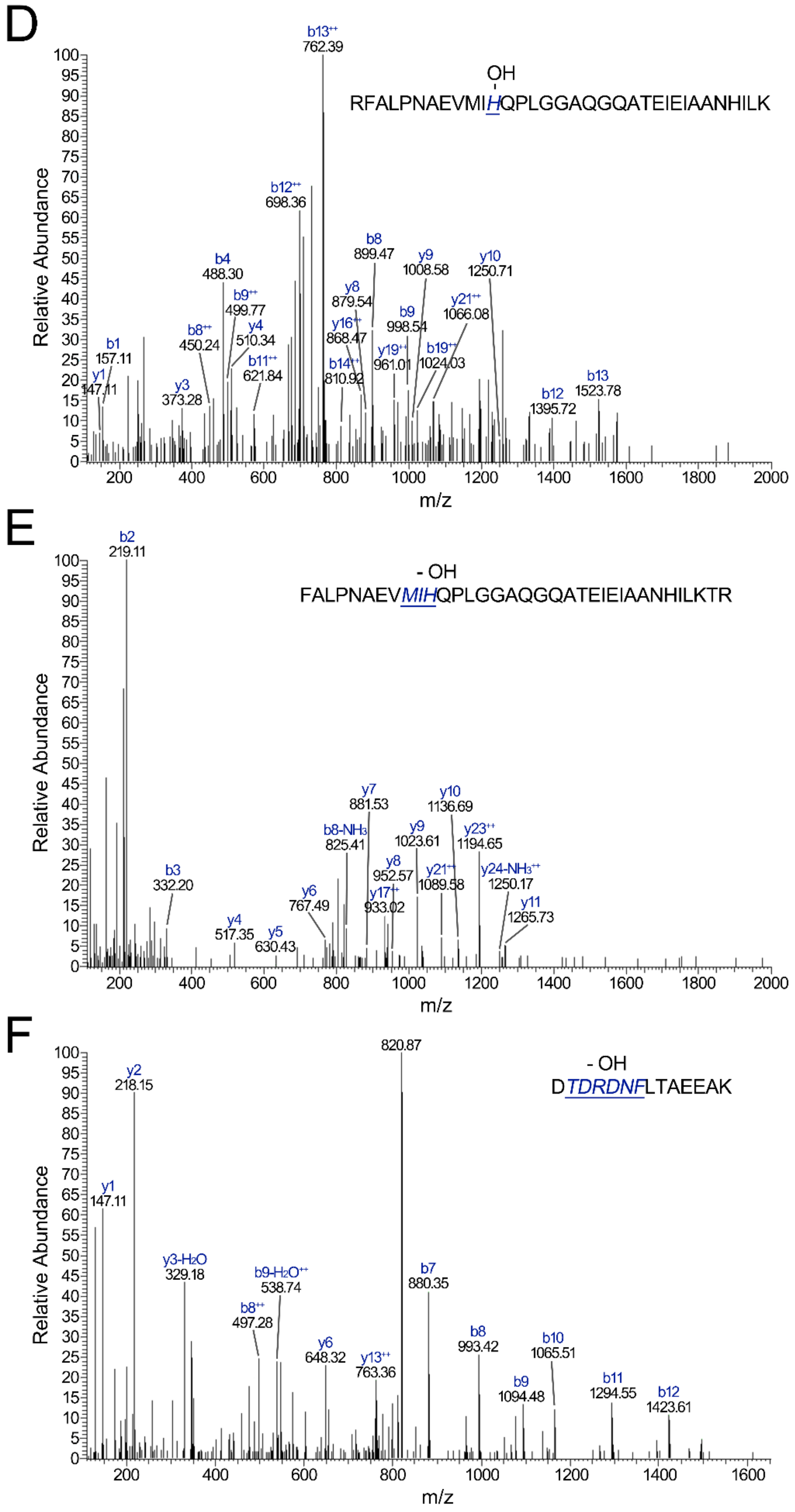
D
OH
RFALPNAEVMIHQPLGGAQGQATEIEIAANHILK
Relative Abundance
m/z
b13++ 762.39
b12++ 698.36
b4 488.30
b9++ 499.77
b8++ 450.24
y4 510.34
b11++ 621.84
b1 157.11
y1 147.11
y3 373.28
b8 899.47
y8 879.54
y16++ 868.47
b14++ 810.92
y19++ 961.01
b9 998.54
y9 1008.58
b19++ 1024.03
y21++ 1066.08
y10 1250.71
b12 1395.72
b13 1523.78
E
- OH
FALPNAEVMIHQPLGGAQGQATEIEIAANHILKTR
Relative Abundance
m/z
b2 219.11
b3 332.20
y4 517.35
y5 630.43
y6 767.49
b8-NH3 825.41
y7 881.53
y17++ 933.02
y8 952.57
y9 1023.61
y21++ 1089.58
y10 1136.69
y23++ 1194.65
y24-NH3++ 1250.17
y11 1265.73
F
- OH
DTDRDNFLTAEEAK
Relative Abundance
m/z
820.87
y2 218.15
y1 147.11
y3-H2O 329.18
b9-H2O++ 538.74
b8++ 497.28
y6 648.32
y13++ 763.36
b7 880.35
b8 993.42
b9 1094.48
b10 1065.51
b11 1294.55
b12 1423.61

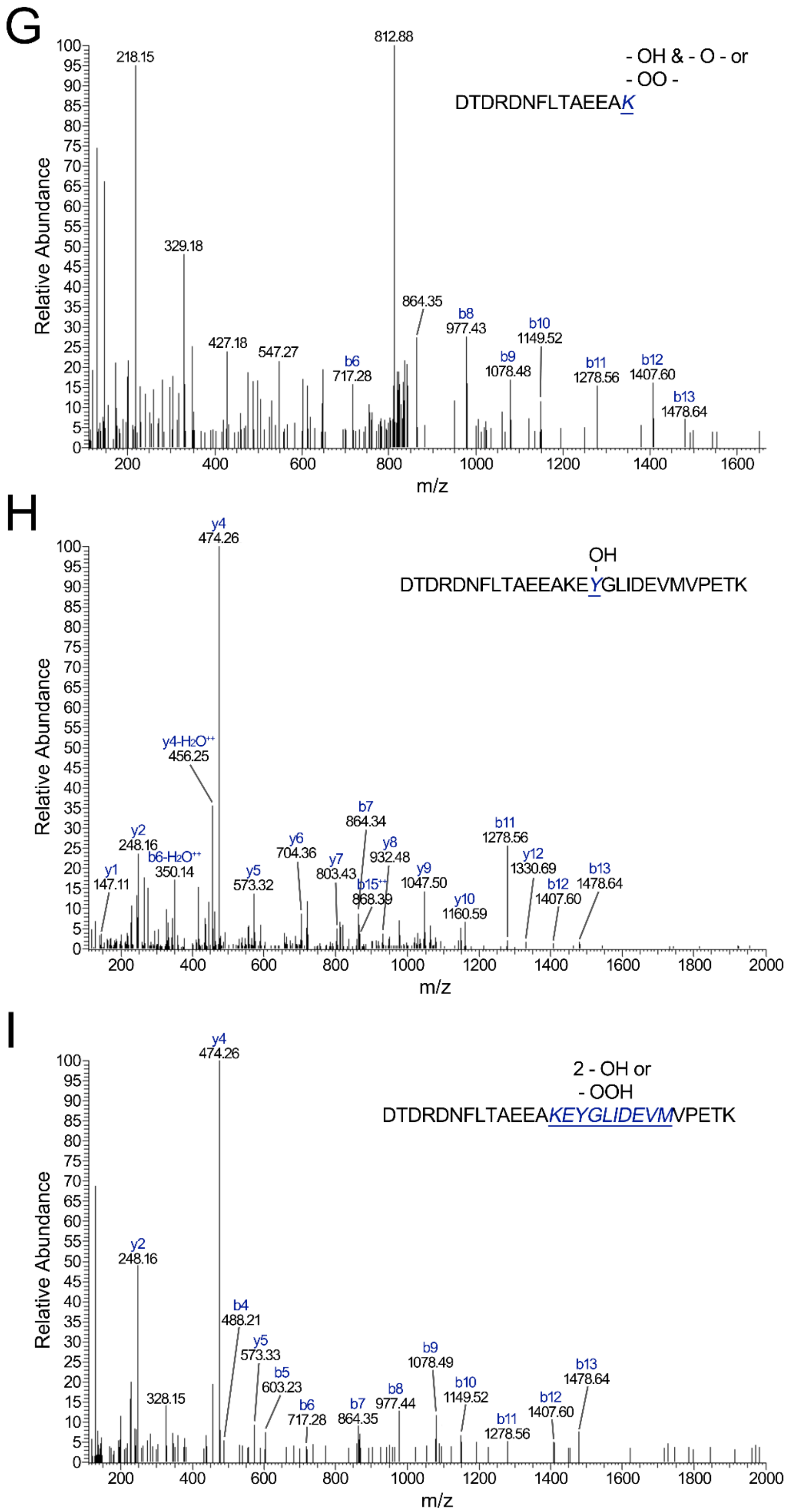

G
- OH & - O - or
- OO -
DTDRDNFLTAEEAK
Relative Abundance
m/z
218.15
812.88
329.18
427.18
547.27
b6 717.28
864.35
b8 977.43
b9 1078.48
b10 1149.52
b11 1278.56
b12 1407.60
b13 1478.64
H
OH
DTDRDNFLTAEEAKEYGLIDEVMVPETK
Relative Abundance
m/z
y4 474.26
y4-H2O++ 456.25
y2 248.16
b6-H2O++ 350.14
y1 147.11
y5 573.32
y6 704.36
y7 803.43
b7 864.34
b15++ 868.39
y8 932.48
y9 1047.50
y10 1160.59
b11 1278.56
y12 1330.69
b12 1407.60
b13 1478.64
I
2 - OH or
- OOH
DTDRDNFLTAEEAKEYGLIDEVMVPETK
Relative Abundance
m/z
y4 474.26
y2 248.16
328.15
b4 488.21
y5 573.33
b5 603.23
b6 717.28
b7 864.35
b8 977.44
b9 1078.49
b10 1149.52
b11 1278.56
b12 1407.60
b13 1478.64
